# Biomechanical Evaluation of Clival Screw Fixation for Occipitocervical Instablity: A Finite Element Analysis

**DOI:** 10.1111/os.14314

**Published:** 2024-12-19

**Authors:** Weipeng Lin, Jianying Zheng, Meichao Zhang, Panjie Xu, Hang Xiao, Wei Ji

**Affiliations:** ^1^ Department of Orthopaedics Yunfu People's Hospital Yunfu China; ^2^ Division of Spinal Surgery, Department of Orthopaedics, Nanfang Hospital Southern Medical University Guangzhou China; ^3^ Department of Anatomy Southern Medical University Guangzhou China

**Keywords:** anterior internal fixation, biomechanics, finite element analysis, occipitocervical fusion, upper cervical spine

## Abstract

**Objective:**

The clivus is trapezoidal in shape with uneven bone structure, the optimal number and position of screws for clival fixation are not clear. Therefore, this study aims to explore the optimization clival screw fixation method for occipitocervical instability using finite element analysis.

**Methods:**

Seven finite element models were developed to evaluate biomechanical properties of clival screw fixation for treating occipitocervical stability, including (i) one clival screw fixation A1 and A2 models; (ii) two clival screws fixation B1 and B2 models; (iii) three clival screws fixation C1 and C2 models; (iv) four clival screws fixation D1 model. Loads of 1.5 Nm were applied to the model fRoM different directions to induce flexion, extension, lateral bending, and axial rotation movements.

**Results:**

The regular triangle C1 type three clival screws fixation exhibited great stability, with RoM of 4.20° in flexion, 5.80° in extension, 0.85° in lateral bending, and 1.60° in axial rotation. The peak stress on the internal fixation devices were relatively low, with maximum screw stress of 194 MPa in flexion, 276 MPa in extension, 180 MPa in lateral bending, and 213 MPa in axial rotation; the maximum plate stress were 126, 554, 426, and 378 MPa, respectively. The areas with higher stress were mainly concentrated at the robust neck section of the plate.

**Conclusion:**

The triangular configuration of three clival screws fixation represented the optimized anterior occipitocervical fixation method through the clivus, offering superior biomechanical stability, lower stress on the devices and dispersed stress distribution in the occipitocervical region.

## Graphical abstract

The clival screws fixation can achieve strong biomechanical stability in the occipitocervical region, which may serve as an optimized occipitocervical fixation method through the clivus.

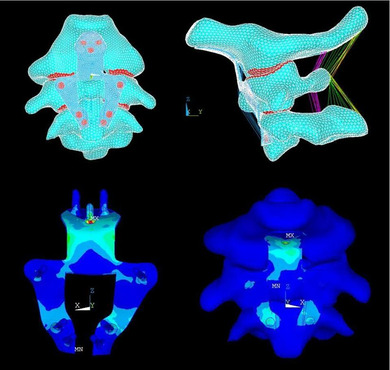

## 1 Introduction

Occipitocervical instability refers to a condition in which the structure and function of the occipitocervical region are compRoMised, leading to a range of clinical symptoms, which may even pose a serious threat to the lives of patients [1]. The posterior approach is currently considered the main treatment for occipitocervical instability. However, in some cases, posterior surgery may not be suitable or cannot be completed, and anterior occipitocervical fixation need to be considered for treatment. For example, in cases such as severe basilar invagination accompanied by atlantoaxial dislocation requiring anterior decompression and fixation [2, 3], anterior upper cervical spine tumors or infections necessitating stability reconstruction [4], and difficulties in achieving posterior fixation due to bone deficiencies [5].

In previous studies, we proposed clival screw fixation for occipitocervical instablity, and designed and invented a clival fixation plate [6]. Subsequently, the feasibility and mechanical stability of clival screw fixation technique were confirmed through a series of anatomical and biomechanical studies [7, 8, 9, 10]. However, researchers reported that following anterior heteromorphic titanium cage reconstruction after resection of upper cervical tumors, screw loosening, extraction, and titanium cage displacement have occurred after single‐screw or double‐screw clival palte fixation. Second, the upper end of the clivus is thicker and dominated by cancellous bone, while the lower end is thinner and primarily composed of cortical bone. There are significant differences in the composition and distribution of cortical and cancellous bone in the clivus, which have a considerable impact on the mechanical properties of screw fixation. Lastly, there is currently no finite element study on the biomechanics of anterior occipitocervical internal fixation via the clivus, and the influence of screw placement locations and quantities in the clivus on the mechanical stability remains unclear.

Therefore, the current study aims to: (i) develop finite element models for occipitocervical instability using clival screw fixation, (ii) evaluate and explore the optimization clival screw fixation method for occipitocervical instability, (iii) provide new approaches for treating occipitocervical instability.

## 2 Methods

### 2.1 Design of Clival Plates

**FIGURE 1:**
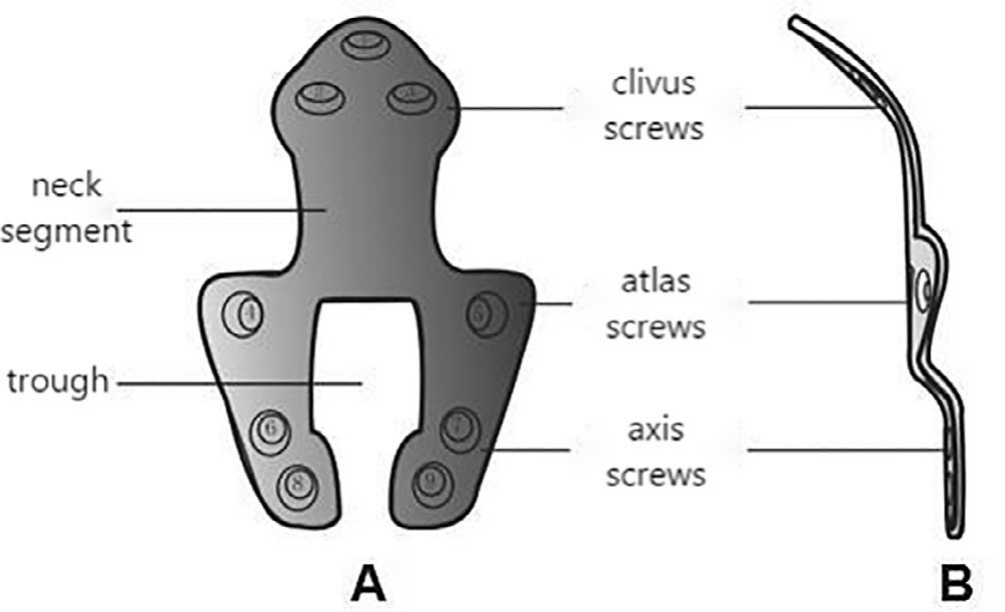
Design of clival plate, which includes three main components: the clival fixation part, the atlas fixation part, and the axis fixation part, with neck section in the middle.

The contour of the clival plate is depicted in Figure 1, which comprises three main components: the clival fixation part, the atlas fixation part, and the axis fixation part. The clival fixation part, measuring 15 mm in height and 20 mm in width, features screw holes for clival screw fixation. The angle between the clival fixation part and the main body of the plate ranges fRoM approximately 114°–148°. The atlas fixation part consists of a neck section that is 15 mm wide and 15 mm high, extending downward fRoM the clival fixation part, and a shoulder section shaped like an inverted T, with an upper width of 35–40 mm, a lower width of 30–35 mm, and a height of 10 mm, extending downward fRoM the neck section. The shoulder section has screw holes on both left and right sides, with screws inserted at an angle of 10°–15° outward into the mass of the atlas, and a groove is formed at the center of the shoulder section. The axis fixation part extends downward fRoM the shoulder section of the atlas fixation part. The axis fixation part has two screw holes for axis fixation on the left and right sides, which securely fixate with the C2 vertebral body via screws. The width of the axis fixation part is approximately 30–35 mm at the top and 15–20 mm at the bottom, with a height of 15 mm. The specific dimensions of the clival plate can be tailored to accommodate individual variations in patients. The screws are designed as 3.5 mm diameter locking screws to effectively prevent screw backout. The material for the plate is titanium alloy (Ti6Al4V), with a thickness of 1.6 mm.

**FIGURE 2:**
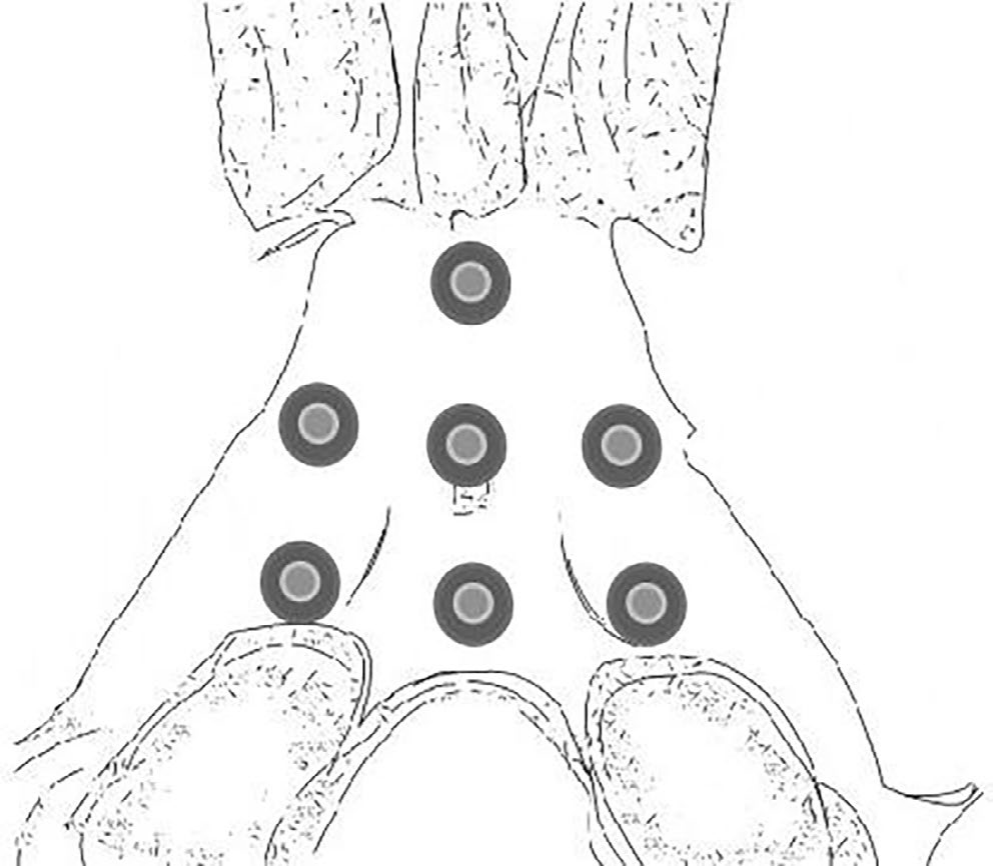
Distribution of placement points for clival screws.

**FIGURE 3:**
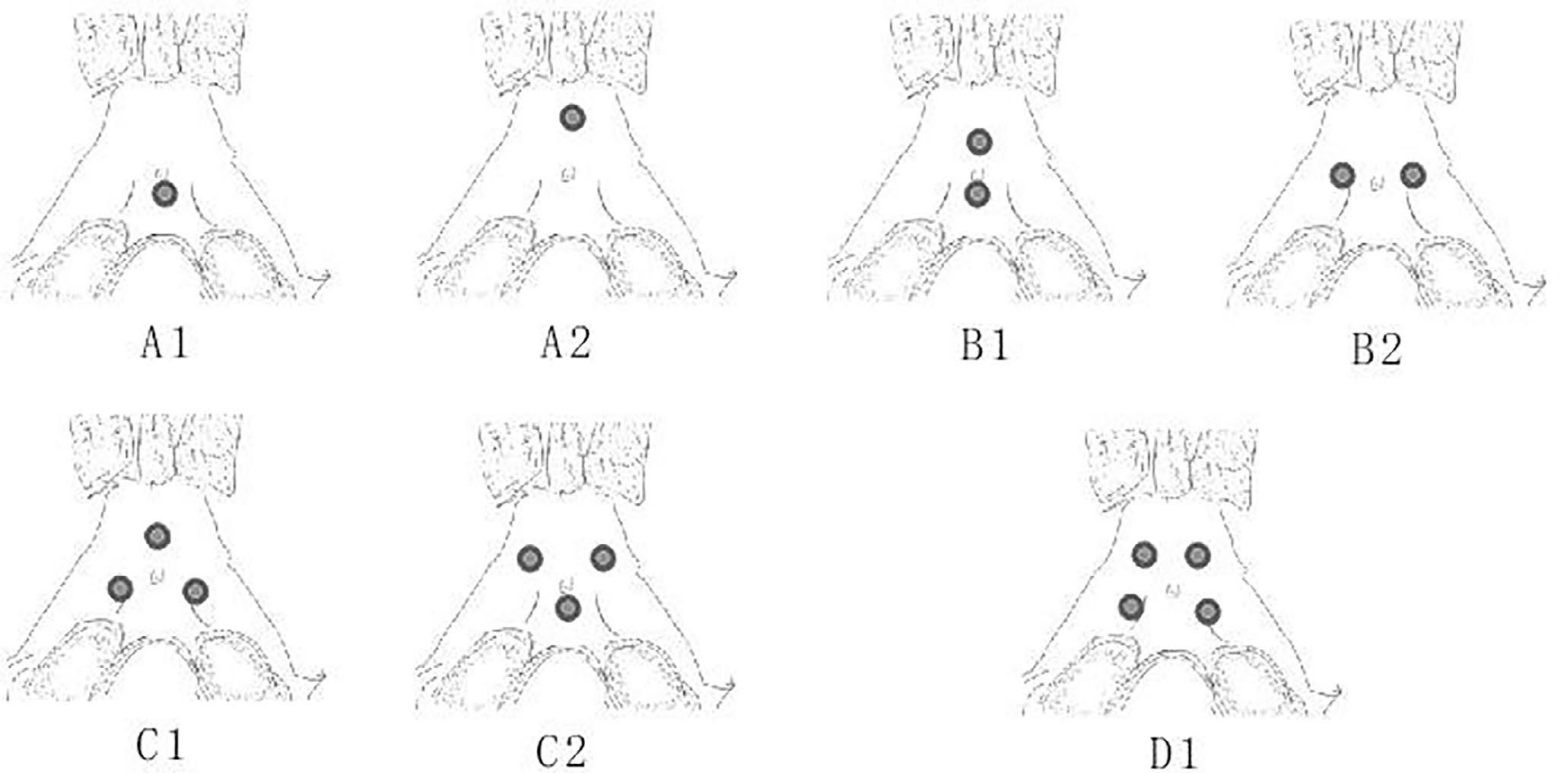
Design of clival screws placement, includes: One screw fixation: A1 type, fixation at the lower parts of the clivus; A2 type, fixation at the upper parts of the clivus. Two screws fixation: B1 type, fixation at the upper and lower points; B2 type, fixation at the left and right points. Three screws fixation: C1 type, equilateral triangular fixation; C2 type, inverted triangular fixation. Four screws fixation: D1 type, trapezoidal fixation.

In this study, the clival fixation part was designed as seven types based on our previous anatomical and morphometric study, as shown in Figures 2 and 3. These types include:

1. One screw fixation: A1 type, fixation at the lower parts of the clivus; A2 type, fixation at the upper parts of the clivus.
2. Two screws fixation: B1 type, fixation at the upper and lower points; B2 type, fixation at the left and right points.
3. Three screws fixation: C1 type, equilateral triangular fixation; C2 type, inverted triangular fixation.
4. Four screws fixation: D1 type, trapezoidal fixation.

### 2.2 Finite Element Model of the Upper Cervical Spine

A healthy male subject aged 36 years, with a height of 171 cm and a weight of 66 kg, was recruited fRoM Nanfang Hospital, Southern Medical University. The subject reported no history of upper cervical spine diseases and surgeries. A routine anteroposterior and lateral cervical X‐ray examination was performed to exclude pathological conditions such as deformities, fractures, tuberculosis, and tumors. A thin‐slice CT scan of the cervical spine was conducted utilizing a German SIEMENS 64‐slice spiral CT scanner with the following scanning parameters: 100 kV, 400 mA, and a slice thickness of 1.00 mm. The thin‐slice CT data were saved in DICOM format. The DICOM files were imported into Mimics 21.0 software (Materialise, Leuven, Belgium) for three‐dimensional reconstruction, and a preliminary three‐dimensional mesh model was established accordingly. The Geomagic 2021 software (Geomagic Sensable group, Wilmington, MA, USA) was used for noise reduction, smoothing, and other surface treatments to optimize the structure of the three‐dimensional mesh model. The model was then imported into SolidWorks 2020 software (Dassault Systemes, Paris, French) for component assembly, detail optimization, and other processing. Finally, the model was imported into ANSYS 14.5 software (ANSYS Inc. Canonsburg, PA, USA) for mesh division and the assignment of material properties to each component, ultimately establishing a three‐dimensional bony finite element model.

The model comprises 130,611 elements and 208,505 nodes, which utilize eight‐node tetrahedral elements to simulate all bone structures and the transverse ligament, while other ligaments are modeled using two‐node cable elements. The remaining tissues are simulated using automatic mesh generation. In the bone structures, the cortical bone has an elastic modulus of 10,000 MPa and a Poisson's ratio of 0.25. Meanwhile, the cancellous bone has an elastic modulus of 4500 MPa and a Poisson's ratio of 0.3. The articular surfaces are reconstructed with articular cartilage, with an elastic modulus of 0.5 MPa and a Poisson's ratio of 0.3. These surfaces exhibit frictionless sliding contact relationships, with a friction coefficient set to 0.1. The transverse ligament, which serves as the primary ligament to maintain the stability of the atlantoaxial joint, is simulated using eight‐node tetrahedral elements, with a length of 20 mm, a width of 8 mm, and a thickness of 3 mm, having an elastic modulus of 20 MPa, and a Poisson's ratio of 0.3. The model's material parameters are assigned based on the properties found in relevant literature [11, 12]. The specific material parameters are presented in Table 1.

**TABLE 1:** Material properties used for various components of the model.

Material	Young's modulus (Mpa)	Poisson's ratio	Cross sectional area (mm^2^)
Bone			
Cortical bone	10,000	0.25	—
Cancellous bone	450	0.3	—
Articular cartilage	0.5	0.3	—
Ligaments			
Transverse ligament	20	0.3	—
Anterior longitudinal ligament	30	0.3	6.1
Posterior longitudinal ligament	20	0.3	5.4
Ligamenta interspinalia	8	0.3	13.1
Ligamentum nuchae	20	0.3	46.6
Ligamentum flavum	9	0.3	50.1
Ligamentum cruciatum	20	0.3	3.6
Alar ligament	7	0.3	22.0
Capsular ligament	8	0.3	5.0
Odontoid ligament	8	0.3	5.0
Devices			
Titanium alloy of plates and screws	113,000	0.3	—

The RoM of the lower surface of the C2 vertebral body in all directions are defined as zero, and a 40 Newton vertical downward force is applied to the occipital condyle surface to simulate the gravitational force of the head. Subsequently, a load of approximately 1.5 Nm is applied to the model fRoM different directions to generate flexion, extension, lateral bending, and axial rotation movements without inducing any injury. The RoM of the normal model at C0‐C1 and C1‐C2 segments under various conditions were calculated and recorded. The results were compared against the biomechanical experimental outcomes of cadaveric samples conducted by Panjabi et al. [13, 14] and the test results of the finite element model developed by Cai et al. [15] to validate the effectiveness of this model. The specific data for the validity verification can be found in Table 2.

**TABLE 2:** Validation of the finite element model of the normal occipitocervical junction (°).

ROMs	Panjabi et al.	Cai et al.	Normal model
C0‐C1			
Flexion	3.5 ± 0.6	3.1	3.2
Extension	21.9 ± 1.9	20.5	19.4
Lateral bending	5.6 ± 0.7	5.1	6.3
Axial rotation	7.9 ± 0.6	7.6	7.6
C1‐C2			
Flexion	11.5 ± 2.0	11.7	11.9
Extension	10.9 ± 1.1	9.5	8.9
Lateral bending	4.0 ± 0.8	4.1	3.9
Axial rotation	38.3 ± 1.7	38.7	36.1

### 2.3 Finite Element Model With Seven Clival Plates

**FIGURE 4:**
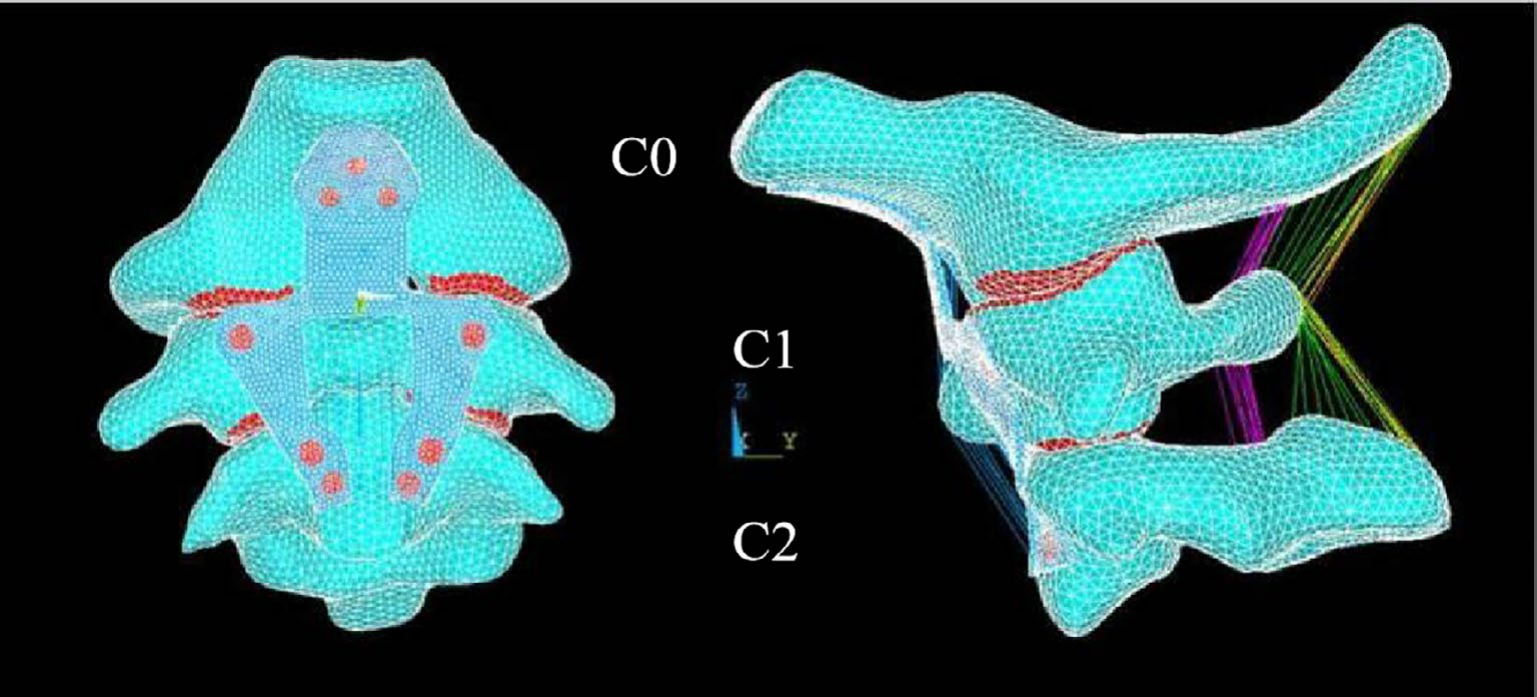
Mesh and stress analysis of finite element model of anterior occipitocervical fixation with clival plate: Front view and side view.

Based on the normal model, elements of the transverse ligament, anterior, and posterior atlantooccipital membranes were manually removed to disrupt the stability of the occipitocervical region, thereby establishing an unstable finite element model. The results were compared and analyzed with those of Cai et al. [15, 16] to verify its validity. On account of the seven types of clival plates, geometric models were established using Mimics 21.0. The geometric models were imported into ANSYS 14.5 as IGES format files for generating three‐dimensional finite element models. The established finite element model is depicted in Figure 4. On the basis of the unstable model, the aforementioned seven types of plates were loaded to obtain seven finite element models with clival plates. The plate was placed in front of the atlas‐axis vertebrae and was closely fitted to the clivus. The entry point of the clival screws was set at the lower 1/3 of the clivus, and the screws were inserted perpendicular to the clivus bone surface until they reached the inner edge of the opposite cortical bone. The entry point of the atlas screws was set at the center of the atlas lateral mass, and the screws were inserted at an angle of 10° outward fRoM the lateral sides of the plate's holes. The entry point of the axis screws was set at the junction of the midline of the axis vertebral body and the lower part of the superior articular process, with the screws inserted in a direction parallel to the upper edge of the axis vertebral body.

### 2.4 Boundary and Loading Conditions

The range of motion of the lower surface of the C2 vertebral body in all directions for the seven types of clival plate finite element models established above was set as zero. A 40 Newton vertical downward force was applied to the surface of the occipital condyle to simulate the gravitational force of the head. Subsequently, a load of approximately 1.5 Nm was applied to the model in the directions of flexion, extension, lateral bending, and axial rotation to produce the corresponding movements without inducing any injury. The RoM of atlanto‐occipital joint, peak stress, and stress nephograms of seven types of clival plate finite element models were calculated and recorded. Stress nephograms were analyzed and compared, and the maximum stress at the screws and plates was calculated using a quantitative method. In this finite element analysis, the interfaces between the screws, plates, and the surrounding bone were considered to be tight contacts with no relative movements (The normal model and the unstable model developed in this study have been validated in the previous section. There is no occipitocervical fixation finite element model similar to this study for validation at this time).

## 3 Results

### 3.1 RoM of Seven Fixation Models

**FIGURE 5:**
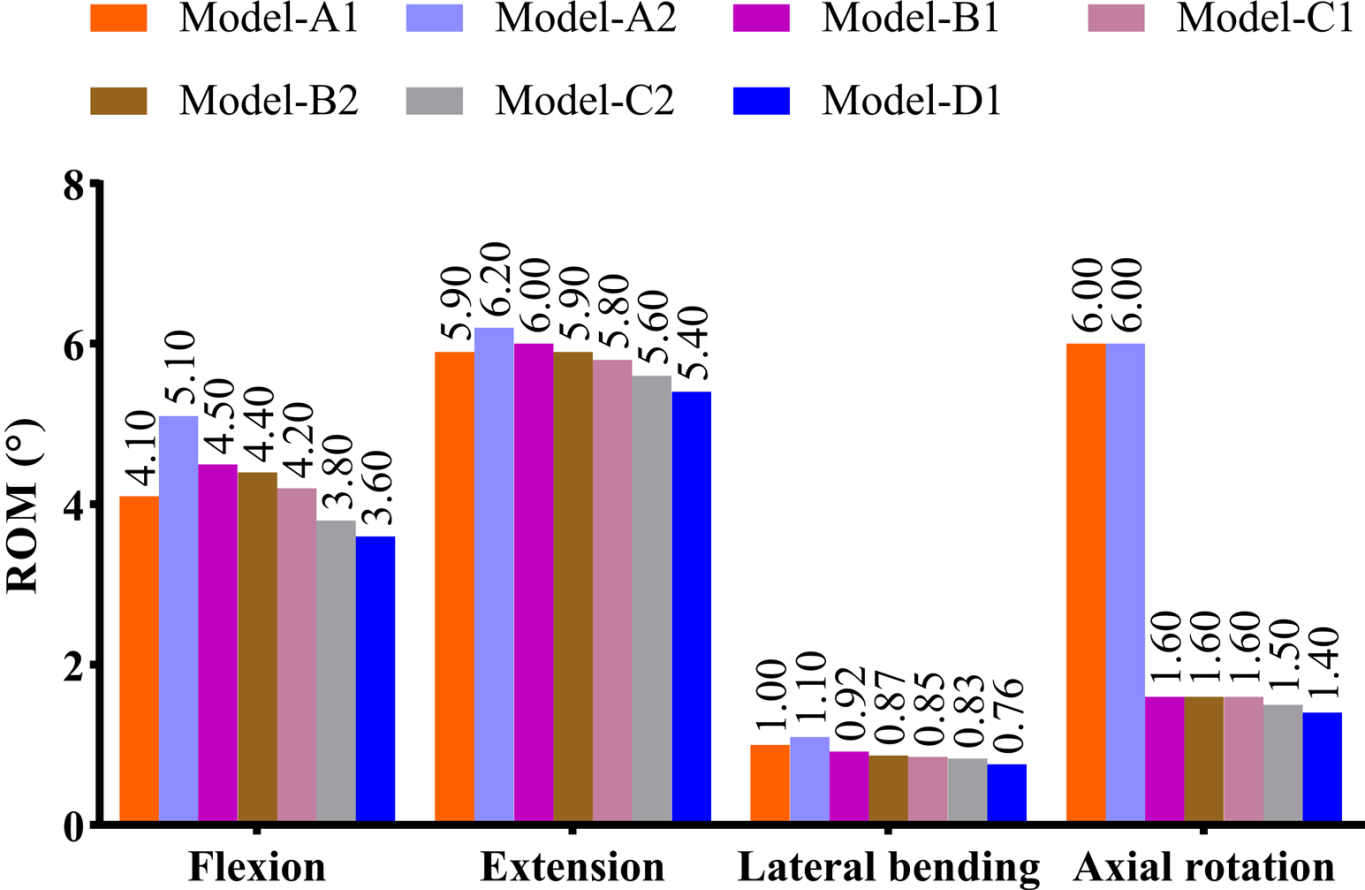
Range of motion of seven finite element models (°).

The RoM of the seven fixation models under different conditions are shown in Table 3 and Figure 5. Compared with the normal model, the RoM of all models showed little difference in flexion, while there was a reduction to varying degrees in other conditions. Compared with the unstable model, the RoM of all models were significantly reduced under each condition. This indicates that the implantation of the plates has achieved satisfactory stability in the occipitocervical region. Among them, the RoM of the C1 type clival plate model were relatively reduced compared to the other six models, with values of 4.20°, 5.80°, 0.85°, and 1.60° under flexion, extension, lateral bending, and axial rotation, respectively.

**TABLE 3:** Validation of the finite element model of the unstable occipitocervical junction (°).

ROMs	Cai et al.	Unstable model
C0‐C1		
Flexion	6.7	6.3
Extension	21.2	25.2
Lateral bending	6.1	7.1
Axial rotation	9.5	7.8
C1‐C2		
Flexion	14.2	15.3
Extension	12.7	12.1
Lateral bending	6.2	5.5
Axial rotation	45.1	51.1

### 3.2 Von Mises Stress of Screws and Plates in Seven Fixation Models

**FIGURE 6:**
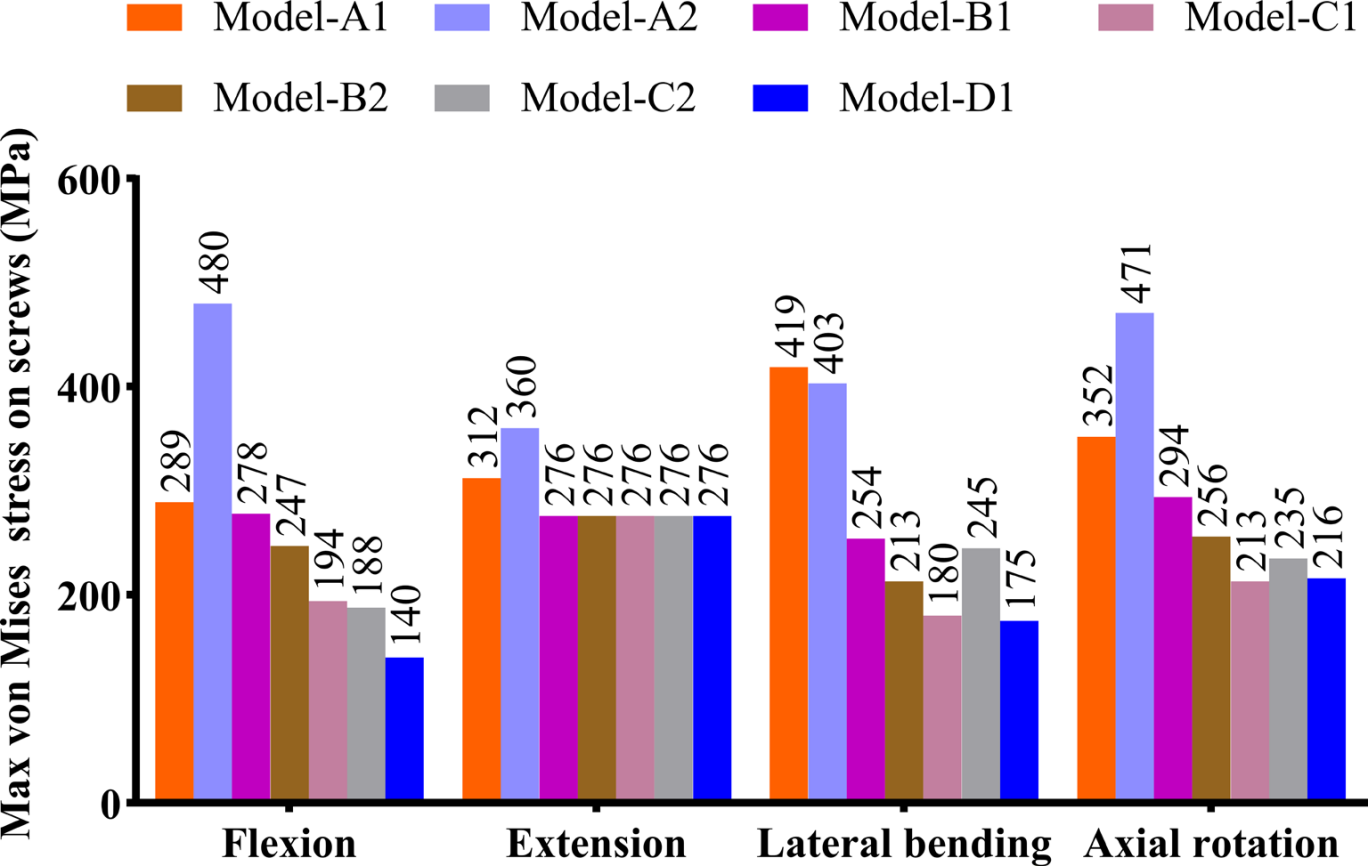
Maximum von mises stress on screws of seven finite element models (Mpa).

**FIGURE 7:**
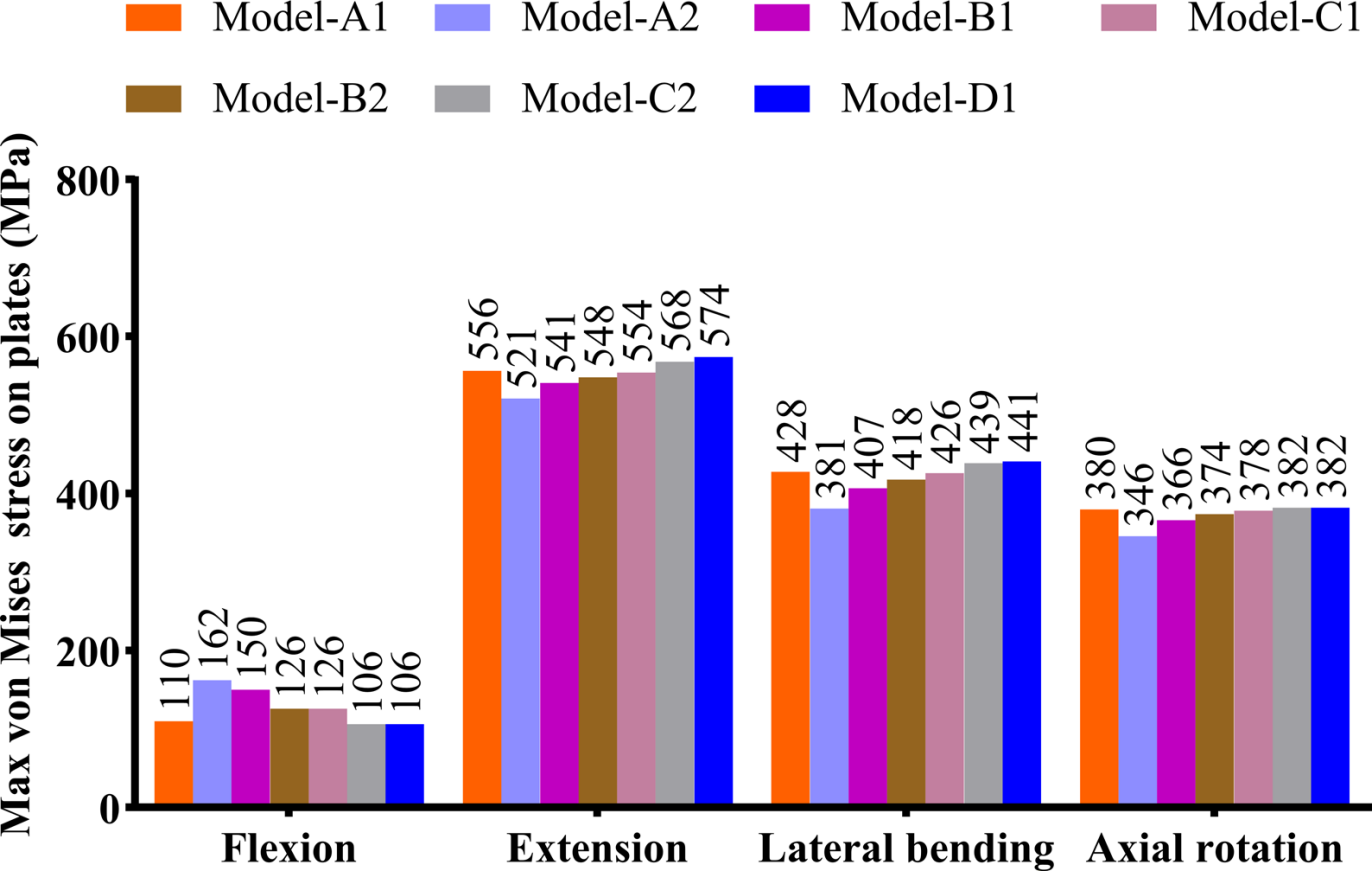
Maximum von mises stress on plates of seven finite element models (Mpa).

The peak stress experienced by the screws and plates in seven fixation models under different conditions are shown in Table 4, Figures 6 and 7. Among them, the C1 type clival plate model exhibited relatively lower peak stress on both the screws and plates. The peak stress under conditions of flexion, extension, lateral bending, and axial rotation are 194, 276, 180, and 213 MPa, respectively for the screws; and 126, 554, 426, and 378 MPa, respectively for the plates.

**TABLE 4:** ROMs of seven finite element models of clival plate fixation (°).

ROMs of C0‐C1	Flexion	Extension	Lateral bending	Axial rotation
Model‐A1	4.1	5.9	1.0	6.0
Model‐A2	5.1	6.2	1.1	6.0
Model‐B1	4.5	6	0.92	1.6
Model‐B2	4.4	5.9	0.87	1.6
Model‐C1	4.2	5.8	0.85	1.6
Model‐C2	3.8	5.6	0.83	1.5
Model‐D1	3.6	5.4	0.76	1.4
Normal model	3.2	19.4	6.3	7.6
Unstable model	6.3	25.2	7.1	7.8

### 3.3 Stress Distribution of Seven Fixation Models

**FIGURE 8:**
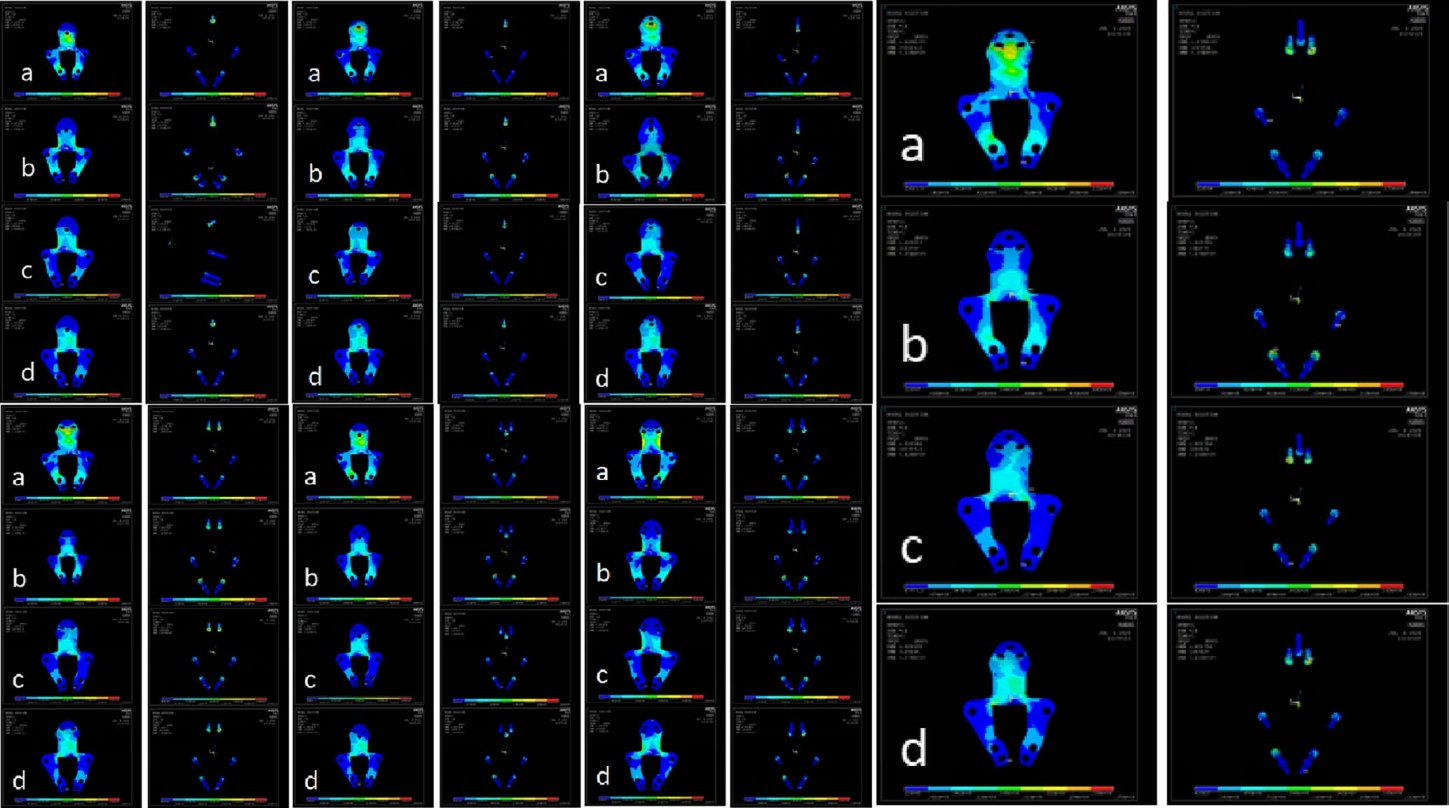
Stress nephograms of clival plate in different directions (a) flexion, (b) extension, (c) lateral bending, (d) axial rotation.

The stress nephograms of seven fixation models are shown in Figure 8. Analysis reveals that the stress levels of the seven types of plates under physiological loading are all within a safe range, primarily concentrated at the junction between the screw‐plate interface. Among them, the C1 type clival plate model exhibits a more dispersed distribution of stress on both the screws and plates. The areas with higher stress are concentrated near the midline of the cervical segment of the plate and at the screw‐plate interface at the bottom of the plate, indicating great resistance to mechanical fatigue (Table 5).

**TABLE 5:** Maximum von mises stress on screws and plates of seven fixation models (MPa).

Maximum von mises stress	Flexion		Extension		Lateral bending		Axial rotation	
	Screws	Plates	Screws	Plates	Screws	Plates	Screws	Plates
Model‐α1	289	110	312	556	419	428	352	380
Model‐α2	480	162	360	521	403	381	471	346
Model‐β1	278	150	276	541	254	407	294	366
Model‐β2	247	126	276	548	213	418	256	374
Model‐γ1	194	126	276	554	180	426	213	378
Model‐γ2	188	106	276	568	245	439	235	382
Model‐δ1	140	106	276	574	175	441	216	382

## 4 Discussion

### 4.1 Main Findings of This Study

This study designed seven types of clival plates and utilized finite element models to optimize the position and quantity of screws on the plates. The range of motion, peak stress and stress distribution on the screws and plates under various conditions were comprehensively analyzed and recorded, finally identified the C1 model as the most optimized method for anterior occipitocervical fixation. This lays the foundation for the clinical application of this internal fixation device, which could serve as a supplementary or alternative method for occipitocervical fixation.

### 4.2 Characteristics of the Finite Element Models

This study successfully established a finite element model of the normal adult occipitocervical region using Mimics 21.0, Geomagic 2021, Solidworks 2020, and ANSYS 14.5 software. The osseous structures, including the joint capsules, were simulated using eight‐node tetrahedral elements, while the ligament structures were modeled with two‐node cable elements. In the osseous structures, the cortical bone had an elastic modulus of 10,000 MPa and a Poisson's ratio of 0.25; the cancellous bone had an elastic modulus of 4500 MPa and a Poisson's ratio of 0.3. All articular surfaces in the model were reconstructed with articular cartilage, with an elastic modulus of 0.5 MPa and a Poisson's ratio of 0.3, which exhibited frictionless sliding contact relationships with a friction coefficient set to 0.1. Additionally, this experiment also manually delineated and reconstructed relevant ligaments based on the anatomical characteristics of the normal occipitocervical region, ensuring that the established finite element model could more truly and accurately reflect the biomechanical characteristics of the actual adult occipitocervical region [16]. Among them, the transverse ligament, as the main ligament maintaining the stability of the atlantoaxial joint, was simulated using eight‐node tetrahedral elements, with a length of 20 mm, a width of 8 mm, a thickness of 3 mm, an elastic modulus of 20 MPa, and a Poisson's ratio of 0.3 [17]. To more accurately simulate the normal upper cervical spine anatomical structure and physiological motion state of the human body, the modeling process as much as possible refined the mesh division. Without significantly affecting the computation speed, the model was made more detailed to obtain more accurate results. The final model contained 130,611 elements and 208,505 nodes.

To ensure the accuracy of the finite element test results, validating the established finite element model is crucial. During the modeling process of the cervical spine finite element model, Yoganandan et al. [18] proposed four key points to consider: the anatomical characteristics of the model, the material properties of various parts, the testing boundary and loading conditions, and the validity verification of the model. In the modeling process of this study, DICOM data fRoM thin‐slice CT scans were used to extract the bony outline of the occipitocervical region. Material properties for each part of the model were assigned based on authoritative domestic and foreign literature, while establishing the boundary and loading conditions. The authenticity of the model was verified by comparing it with classic cadaveric biomechanical experiments. Therefore, it can be considered that the finite element model established in this study can effectively simulate the anatomical structure and motion state of the occipitocervical region. Its finite element test results are true and accurate, thus reflecting the biomechanical characteristics of the adult occipitocervical region.

### 4.3 Characteristics of the Clival Plates

The clival plate is characterized by a triangular design of its clival section, with the apex pointing upward, which is designed to better match the physiological anatomical form of the clival area. By securing multiple screws in this area, the plate can closely conform to the outer cortical bone of the clivus, thereby making it less likely to loosen or cause bone damage. Additionally, based on the anatomical data related to the clivus obtained by Ji et al. [6], the dimensions (length, width, height, and thickness) of the plate are reasonably set to enable internal fixation within the safe range of the clivus. The atlas section of the plate, where it is widest, can closely adhere to the surface of the atlas lateral mass and provide a larger range of screw insertion angles to meet the needs of different clinical situations. To accommodate the anatomical form of the axis vertebra, the axis section of the plate is concave inward compared to the atlas section and is equipped with four screw holes, distributed at the junction of the midline of the axis vertebral body and the lower part of the superior articular process, ensuring the strength and surgical safety of internal fixation in this area. The plate is designed with a thickness of 1.6 mm, which is thinner than the previous 2 mm anterior plate for the atlas‐axis, aimed at minimizing the tension of the posterior pharyngeal wall suture postoperatively, accelerating wound healing, and further reducing the probability of infection and exposure of the internal fixation device.

There are many important nerves, blood vessels, tissues, and organs located around the occipitocervical region. To reduce the risk of iatrogenic injury caused by internal fixation, the entry point for the atlas screw is set at the center of the atlas lateral mass, with a total of two screws inserted, adhering to the “safe zone” for transoral screw insertion into the atlas proposed by Kandziora et al. [19] The entry point for the axis screw is set at the junction of the midline of the axis vertebral body and the lower part of the superior articular process, with a total of four screws inserted. After studying the anatomy of the clival, Ji et al. [6] found significant differences in the composition and distribution of the cortical and cancellous bone at the upper and lower ends of the clival, which greatly affect the mechanical performance of the bone screw fixation. Therefore, the entry point for the clival screw is set at the lower one‐third of the clival, with a total of three screws inserted. Subsequently, after biomechanical evaluation, it was confirmed that the clival plate can achieve good screw fixation strength and surgical safety.

However, the effect of the number of screws in the clival area on the overall stability of anterior occipitocervical internal fixation is still unclear. A greater number of screws necessitates more extensive anatomical exposure and dissection during surgery, greatly increasing the risk of injury to surrounding important structures, particularly the internal carotid artery running through the foramen lacerum. Furthermore, the transoral surgical approach is difficult or even impossible to achieve the insertion of screws at higher positions of the clival (insertion of screws at the upper end of the clival may require a mandibular split approach). Whether a smaller number of clival screws can achieve the equivalent stability of occipitocervical fixation as other methods of screwing also needs further research. Therefore, this study developed seven types of clival plates by changing the number and distribution of screw holes in the clival area. It utilized the finite element analysis method to optimize the number of screws in the clival area and analyzed the stress, strain, and stability around different clival plates and screws to provide a scientific basis for new treatment methods of anterior clival screwing for unstable occipitocervical regions.

### 4.4 Comparison of Mechanical Properties of the Seven Fixation Models

The seven clival plate finite element models significantly reduced the three‐dimensional range of motion in the occipitocervical region after injury under conditions of flexion, extension, lateral flexion, and rotation, all indicating good stability. To improve the mechanical performance of the instrumentation, comparative analysis between groups revealed that the D1 four‐screw clival plate model had the adifferent working conditions. Moreover, the plate stress peak values also had advantages over the other six models, suggesting it could be identified as the most optimized approach. However, observation of the stress Stress nephograms showed that the stress on the plate was concentrated between the neck segment of the plate and the atlas fixation screw hole, making it prone to stress fatigue and may have a higher risk of fracture. Additionally, the stress on the screws was mainly concentrated at the screw hole located below the clival fixation part. Furthermore, increasing the number of screws above the clival would not serve to disperse the stress. Conversely, setting the clival screw entry point higher or more laterally on the bone surface could potentially damage the internal carotid artery, the inferior petrosal sinus, the sphenoid sinus, or even the hypothalamus [6]. Lastly, the D1 type clival plate requires the implantation of four screws in the clival area, which inevitably necessitates a wider dissection of the surrounding tissue, increasing the risk of bleeding, infection, dysphagia, cerebrospinal fluid leakage, and meningitis, thereby being not conducive to postoperative recovery [20, 21].

The C1 three‐screw clival plate model had a three‐dimensional range of motion of 4.20°, 5.80°, 0.85°, and 1.60° under conditions of flexion, extension, lateral bending, and axial rotation, respectively, exhibiting similar mechanical stability to the D1 model. The maximum screw stress under these conditions was 194, 276, 180, and 213 MPa, respectively, which was similar to the D1 model and significantly better than the other five models. The maximum plate stress was 126, 554, 426, and 378 MPa, respectively, which was also similar to the D1 model. Upon observation of the stress Stress nephograms, it was found that the stress on the C1 type plate was mainly concentrated in the stronger neck segment of the plate, and the remaining stress was more dispersed than that of the D1 plate, indicating better resistance to fracture and screw pullout. Furthermore, the C1 type plate involved reduced tissue dissection during the operation, potentially leading to improved clinical outcomes. However, as an anterior transoral pharyngeal approach for internal fixation, it still poses significant risks, and preoperative imaging examination of the clival is essential [19]. In summary, the C1 type clival plate can be determined as the most optimized clival plate in this study, which is consistent with the biomechanical testing conclusions of the clival plate for anterior occipitocervical fixation by Ji et al. [10].

### 4.5 Strengths of This Study

In this study, the DICOM data of thin‐layer CT scan were used to extract the bony contour of the occipitocervical region, refer to the authoritative literature to give the material properties of each part of the model, establish the boundaries and loading conditions, and verify the authenticity of the model by comparing it with the classical cadaveric experiments. Therefore, it can be concluded that the finite element model established in this study can effectively simulate the anatomical structure and motion of the occipitocervical region, and its results are real and accurate, which can well reflect the anatomical structure and biomechanical properties of the occipitocervical region. Moreover, the clival section of the plate is designed as a triangular shape with the tip upward, which can better match the physiological and anatomical morphology of the clivus. By completing multiple screw fixation in this area, the plate can be tightly adhered to the lateral bone cortex of the clivus, which is tough to loosen or cause bone damage. In addition, the length, width, height, and thickness of the plate were rationally set so that it could be internally fixed within the safe range of the clivus. The atlantoaxial section is the widest part of the plate, which can fit well on the surface of the atlantoaxial lateral mass and provide a wide range of screw entry angles to meet the needs of different clinical situations. In order to match the anatomical shape of the atlas, the atlantoaxial section of the plate is more concave than the atlantoaxial section, and 4 screw holes are arranged on both sides of the midline of the vertebral body of the atlas and below the superior articular process, which ensures the strength of internal fixation and surgical safety in this area. The thickness of the plate is 1.6 mm, which is thinner than the previous 2 mm anterior atlantoaxial plate, to minimize the tension of the posterior pharyngeal wall suture, accelerate wound healing, and further reduce the probability of infection and exposure of the internal fixation.

### 4.6 Limitations of This Study

In the other hand, the limitations are as follows: The finite element models developed has realistic anatomical details and highly simulated tissue structure and material properties. However, there are some limitations. First, Finite element analysis utilizes digital models to simulate physiological motion but cannot assess the impact of bone grafting and fusion on stability in actual surgical procedures. Second, FEA may not effectively reflect the changes in the holding force between internal fixation devices and bone quality under various bone conditions, particularly in cases of osteoporosis. Third, FEA simulates the vertebrae, intervertebral discs, and ligaments using linear, elastic, and homogeneous materials, neglecting the role of muscles, which differs fRoM the actual nonlinear structure of the human body. Fourth, this study compares the occipitocervical internal fixation models fRoM different domestic and foreign literature, and the reliability of the comparative results may be affected due to inconsistencies in the data sources and modeling methods. Fifth, in this study, the plate, screws, and bone surface are set as a single entity, neglecting the micro‐motion between the screw‐plate and screw‐bone, which may influence the results of the finite element analysis. Sixth, in clinical practice, the specific size of the plate can be individually designed according to the differences among patients, and variations in size and position may lead to different finite element analysis results.

## 5 Conclusion

The regular triangle C1 type three screws fixation can achieve strong biomechanical stability in the occipitocervical region, with low stress, dispersed stress distribution on the devices, and low risk of fracture and failure. This technique can serve as an optimized occipitocervical fixation method through the clivus. However, additional biomechanical experiments should be performed to verify the reliability of the internal fixation.

## Author Contributions

Wei Ji designed the study and coordinated the resources. Weipeng Lin, Jianying Zhen, and Meichao Zhang performed the research and collected data. Weipeng Lin, Jianying Zheng, and Wei Ji analyzed the results. Jianying Zheng and Hang Xiao drafted the manuscript. Wei Ji and Hang Xiao revised the manuscript. Wei Ji supervised the study.

## Ethics Statement

This study was approved by the Ethic Committee of Nanfang Hospital.

## Conflicts of Interest

The authors declare no conflicts of interest.
